# Traffic noise exposure depresses plasma corticosterone and delays offspring growth in breeding zebra finches

**DOI:** 10.1093/conphys/coz056

**Published:** 2019-10-11

**Authors:** Sue Anne Zollinger, Adriana Dorado-Correa, Wolfgang Goymann, Wolfgang Forstmeier, Ulrich Knief, Ana María Bastidas­Urrutia, Henrik Brumm

**Affiliations:** 1 Department of Natural Sciences, Manchester Metropolitan University, John Dalton East, Chester Street, Manchester M1 5GD, UK; 2 Communication and Social Behaviour Group, Max Planck Institute for Ornithology, Eberhard­Gwinner­Strasse 1, 82319 Seewiesen, Germany; 3 Department of Behavioural Neurobiology, Max Planck Institute for Ornithology, Eberhard­Gwinner­Strasse 1, 82319 Seewiesen, Germany; 4 Department of Behavioural Ecology and Evolutionary Genetics, Max Planck Institute for Ornithology, Eberhard­Gwinner­Strasse 1, 82319 Seewiesen, Germany; 5 Division of Evolutionary Biology, Faculty of Biology, Ludwig Maximilian University of Munich, Grosshaderner Strasse 2, 82152 Planegg­Martinsried, Germany; 6 Biodiversity and Global Change Lab, Terrestrial Ecology Research Group, Technical University of Munich, Hans­Carl­von­Carlowitz­Platz 2, 85354 Freising, Germany

**Keywords:** Anthropogenic noise, glucocorticoids, reproductive fitness, songbird, stress, urbanization

## Abstract

The impact of human activity on the acoustic environment is overwhelming, with anthropogenic noise reaching even remote areas of the planet. The World Health Organization has identified noise pollution as one of the leading environmental health risks in humans, and it has been linked to a myriad of short- and long-term health effects in exposed individuals. However, less is known about the health effects of anthropogenic noise exposure on animals. We investigated long- and short-term effects of traffic noise on zebra finches breeding in small communal aviaries, using a repeated measures design. Birds bred in both noise and no-noise conditions, and we measured baseline plasma glucocorticoid levels before, during and after breeding. In addition, we assayed immune function, measured reproductive success and offspring growth and compared rates of extra-pair paternity of breeding adults. Breeding birds had significantly lower baseline plasma corticosterone levels when exposed to traffic noise than when they were not exposed to noise playback. In addition, the nestlings reared during noise exposure were lighter than nestlings of the same parents when breeding in control conditions. Our results suggest that traffic noise poses a more severe hurdle to birds at more vulnerable stages of their life history, such as during reproductive events and ontogeny. While chronic exposure to traffic noise in our birds did not, by itself, prove to be a sufficient stressor to cause acute effects on health or reproductive success in exposed individuals, it did result in disruptions to normal glucocorticoid profiles and delayed offspring growth. However, animals living in urban habitats are exposed to a multitude of anthropogenic disturbances, and it is likely that even species that appear to be thriving in noisy environments may suffer cumulative effects of these multiple disturbances that may together impact their fitness in urban environments.

## Introduction

Humans have been dramatically altering the environment for millennia, and as the global population swells, urbanization is ever increasing. The growth of urban areas is projected to reach exceptional levels in the coming decades, and this expansion of human developments will continue the fundamental alteration of ecosystems (United Nations, 2014). Some of the challenges that animals face in cities are habitat fragmentation, changes in micro-climate, limitation of resources, alteration of resource flow, changes in species interactions, as well as pollution (Grimm *et al*., 2008; Barber *et al*., 2009; Shanahan *et al*., 2014).

Pollution is one of the main consequences of human development and may take the form of chemical contamination as well as light and noise emissions. The past decade has seen a groundswell of interest in the effects of anthropogenic noise pollution on birds, primarily focused on changes in vocal behaviour. Dozens of studies have reported correlations between background noise levels and various song characteristics. For instance, in response to increases in the background noise levels, birds increase the amplitude of their songs, as well as the redundancy, duration, frequency and the timing of their vocalizations (reviewed in Brumm and Zollinger, 2013). These observations have led many to suggest that these modifications of vocal behaviour are an attempt of birds to ameliorate the negative effects of noise on acoustic communication (Gil and Brumm, 2013), although some tactics are likely to be more effective at mitigating the effects of noise than others (see Nemeth and Brumm, 2010). For signal receivers, noise can have negative fitness consequences by masking important acoustic signals and cues, such as alarm signals, sounds made by predators or prey or signals relevant for breeding behaviour and offspring care (Brumm, 2010; McIntyre *et al*., 2014; Read *et al*., 2014; Templeton *et al*., 2016). Birds may be particularly sensitive to noise disruption during their reproductive period, since many species rely on acoustic signals to attract mates and defend territories, to maintain pair bonds and coordinate offspring feeding and care (Catchpole and Slater, 2008). Thus, acoustic masking by noise is likely to have major fitness consequences (Brumm and Slabbekoorn, 2005).

There is some evidence from correlational studies comparing noisy and less noisy sites, that noise exposure can influence avian reproductive success in different ways. For example, great tits (*Parus major)* breeding in areas with high levels of traffic noise produced fewer fledglings and laid smaller clutches than their conspecifics in quieter areas (Halfwerk *et al*., 2011). Eastern bluebirds (*Sialia sialis)* nesting in noisy areas also had reduced reproductive success, with lower hatching rates and fledging success than their conspecifics at quieter sites (Kight *et al*., 2012). House sparrows (*Passer domesticus*) breeding at a noisy site hatched fewer young, of lower body mass, produced fewer recruits and had lower rates of offspring provisioning than sparrows in quiet sites (Schroeder *et al*., 2012). Noise can also mask parental alarm calls, reducing the response of nestlings towards predator threats (McIntyre *et al*., 2014). Detection of alarm calls by great tits is significantly impaired in the presence of traffic noise (Templeton *et al*., 2016), which is likely to increase the risk of predation in habitats with high levels of noise pollution. In line with the notion of increased predation risk in noise, some species have been shown to increase vigilance time in noise, resulting in a reduction of feeding rate (Fernández-Juricic and Tellería, 2000; Quinn *et al*., 2006), but see (Yorzinski and Hermann, 2016).

Noise can also have negative non-auditory effects, in that it may act as a stressor and could increase physiological stress responses such as elevated plasma glucocorticoids, which can lead to depressed immune function and increased oxidative stress in the brain and organs of the immune system (reviewed in Kight and Swaddle, 2011). Several species of birds have shown correlations between levels of the hormone corticosterone and chronic environmental noise, although the size or direction of these correlations seem to vary by species, life history stage and context (Wright *et al*., 2007; Crino *et al*., 2011, 2013; Blickley *et al*., 2012; Potvin and MacDougall-Shackleton, 2015a; Kleist *et al*., 2018). Elevated levels of corticosterone in mothers led to small body size and slow plumage development in offspring (Saino *et al*., 2005). Elevated corticosterone has also been found to reduce the time parents spend incubating eggs and to increase the instances of nest abandonment (Spée *et al*., 2011; Thierry *et al*., 2013). Parents with high levels of plasma corticosterone may also reduce feeding rates (Angelier *et al*., 2009). On the other hand, in some studies increased levels of corticosterone seemed to help parents adapt to new situations better (Escribano-Avila *et al*., 2013), for example by resulting in an increase of parental care and number of fledglings (Bonier *et al*., 2009, 2011).

To date, most studies on the effects of noise on birds have been conducted in the field. While field studies are important to identify potential relationships between environmental factors and changes in populations, they can present challenges for understanding causal relationships, as there are often many interacting factors that cannot be controlled for, such as chemical pollution (Isaksson, 2010), artificial light at night (Dominoni *et al*., 2016), avian community density and composition (McKinney, 2006), habitiat structure (Nemeth and Brumm, 2010), food type and availability (e.g. Biard *et al*., 2017) and others. To understand the causal effects that underlie observed correlations between anthropogenic noise and changes in behaviour or fitness, it is important to identify model systems that can be experimentally manipulated. Several recent studies have investigated the effects of experimental traffic noise exposure on glucocorticoids and reproductive success in breeding birds in controlled field experiments, and in a range of songbird species, with mixed results (e.g. Angelier *et al*., 2016; Halfwerk *et al*., 2016; Davies *et al*., 2017; Injaian *et al*., 2018). As far as we are aware, just one previous laboratory study has investigated the direct effect of noise on corticosterone levels and reproductive success on captive birds (Potvin and MacDougall-Shackleton, 2015b). This study exposed zebra finches to an artificial mix of noises from cars, trains, motorcycles and lawnmowers and found trends towards increased embryo mortality and reduced nestling growth, although their study had quite low statistical power, and neither of these effects reached significance. They further found no chronic effect of noise exposure on plasma corticosterone levels.

In the present study we experimentally exposed breeding zebra finches (*Taeniopygia guttata*) to realistic levels of recorded traffic noise and measured whether noise exposure alone was sufficient a stressor to affect baseline glucocorticoid levels, immune function, reproductive success or levels of extra-pair paternity. We included extra-pair paternity in our analysis as an earlier study found that very high levels of noise exposure reduced the preference of female zebra finches for their pair-bonded males (Swaddle and Page, 2007), but it is unclear whether more realistic levels of environmental noise pollution would have a similar effect. The experiment was conducted in a pair-wise design in which birds living in communal aviaries were allowed to reproduce twice, once exposed to traffic noise and once without noise playback. We hypothesized that traffic noise would act as chronic stressor. While there appears to be no one way in which different species respond to chronic stress, the majority of species in a recent meta-analysis were found to respond to chronic stress with an increase in baseline glucocorticoids (Dickens and Romero, 2013). Thus, we expected to find higher levels of baseline corticosterone, a weaker immune system and a lower performance in reproductive success, including a negative effect on the size and/or body condition of chicks.

## Materials and methods

### Study system

We allowed 88 adult zebra finches (1–2 years old) from the colonies at the Max Planck Institute for Ornithology in Seewiesen, Germany to breed in 6 aviaries. The birds used for breeding were the naïve offspring of a cross between populations 11 and 18 (Forstmeier *et al*., 2007a), which had been housed prior to our experiment in groups of 4–8 birds in single-sex cages (240 cm long, 40 cm deep and 45 cm high). Each of two experimental rooms contained three aviaries (1 × 2 × 2 m), separated by opaque dividers, housing 7–8 pairs of birds. Each aviary was provided with 12 wooden nest boxes and *ad libitum* nesting materials, seeds, commercial finch egg food and water. In addition, birds were provided with fresh vegetables and hard-boiled eggs twice weekly throughout the experimental period. Animal housing and care was in accordance with European and German laws governing the care and use of laboratory animals (Council of Europe Treaty ETS-123). All experimental procedures were approved by and done under license from the Government of Upper Bavaria (Regierung von Oberbayern), licence number 55.2-1-54-2532-51-2013.

### Experimental treatment

To determine if typical city traffic noise affects baseline plasma corticosterone levels, reproductive success and immune function in adult birds, we used a crossed­design experiment. This means that one half of the aviaries (in Room 1) were treated with traffic noise throughout the first round of breeding, egg laying and nestling care. The other half (in Room 2) was not treated with noise. After the youngest of the offspring of the first round of breeding reached 120 days post-hatch, the offspring were moved out of their natal aviaries into a separate building. After a 30-day pause, a second breeding round started, in which the noise treatment was switched between rooms, that is breeding pairs that were previously exposed to noise were allowed to breed without noise, and breeding pairs that were previously allowed to breed without noise were now exposed to noise.

Daytime noise playbacks consisted of 80, 5-min long recordings of street traffic noise, which were recorded at several busy intersections in and around Munich, Germany during April 2013. Recordings were made along busy four or six lane roads within the city and along six to eight lane motorways. The recordings varied in vehicle type and pass frequency, and all files included passenger cars, with some also including motorcycles, pedestrian and bicyclist noise including shouting, large trucks and emergency vehicles including sirens. During the daylight hours (06:30–20:30), the 80 recordings were played continuously, in randomized order, with playback levels fluctuating between 65 and 85 dB(A) re 20 μPa at the position of the nest boxes. Nighttime playback (20:30–06:30) consisted of randomized playbacks of an additional 40 noise recordings, which were less dense in the rate of passing traffic than the daytime recordings and were reduced in peak amplitudes, with playback level averages fluctuating between 45 and 75 dB(A). None of the nighttime noise files contained sirens or loud small engine revving that characterized some of the daytime files. Noise playback mimicked typical urban noise patterns, according to published noise maps (Bayerisches Landesamt, 2012). During the control condition no noise playback occurred, and the noise levels in the rooms (exclusive of bird sounds) were 40–48 dB(A), depending on the cycling of the ventilation system. We played noise from a laptop computer to an array of 12 pairs of amplified portable speakers (Hama Sonic Mobil 400 Alu PS1032), with 4 pairs arranged above each of the 3 aviaries in the room. Noise playback was run using a script written in MatLab (version 7.5.0; Natick, MA, USA; www.mathworks.com) to randomize playback during day and night. For the noise treatment, playback of noise began 4 weeks before the introduction of nesting materials and nest boxes and continued until the median juvenile in the room had fledged (the date when half of the offspring had fledged, with an average fledgling age of 17 days post-hatch).

### Baseline corticosterone levels

**Figure 1:**
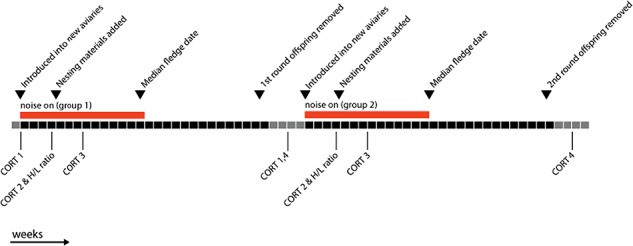
Timeline of the experimental procedure and blood sampling periods. Each block represents 1 week, black blocks are within the experimental reproductive rounds, while grey blocks are ‘rest periods’ during which birds were either housed in single-sex flight cages, or in mixed groups in home aviaries, but not provided with nesting materials.

To determine hormone profiles, we captured birds in the experimental rooms and collected blood samples within 3.5 min of entering the room. Baseline corticosterone levels collected in less than 3 min from the start of the disturbance are not thought to be greatly affected by capture and handling (Romero and Reed, 2005, but see Results). Each bird was sampled four times during the breeding cycle: (i) pre-treatment (before birds were moved into the group aviaries from the single-sex housing in which they were being held prior to our experiment); (ii) during the courtship period; (iii) during the nesting period (incubating eggs, but before hatching); and (iv) post-treatment (2 weeks after the grown offspring were removed from the group aviaries at 120 days post-hatch (10 days after the end of the experiment). Pairs were still together in the group aviaries, but nest boxes had been removed and no nesting materials were provided. Sampling period 4 (post-treatment) from round one, was used as sampling period 1 (pre-treatment) for the second breeding round; see timeline in Fig. 1. All blood samples were collected between 11:00 and 12:00 h. We did not take a blood sample during the nestling care period because during a pilot study we found that catching and bleeding of the parents during this period resulted in chicks that were delayed in somatic growth and increased levels of chick mortality.

### Blood sampling techniques and hormone assays

Blood samples were collected by brachial venipuncture for each bird and were collected into heparinized capillary tubes (1.4 × 75 mm), transferred into Eppendorf tubes and centrifuged with 5000 rpm for 10 min to separate the plasma. Plasma was stored at −80°C. Corticosterone concentrations were determined by radioimmunoassay following Goymann *et al*. (2006). Corticosterone antibodies were obtained from Esoterix Endocrinology, Calabasas Hills, CA. Extraction efficiency (as calculated from tracer amounts of tritiated hormone added to each sample before extraction) was 83.2 ± 4.9% (*N* = 266) for 3H-corticosterone (Perkin Elmer, NET 399). The average limit of detection ranged from 2.94 to 3.39 pg corticosterone per tube. We ran a total of five corticosterone assays and the intra-assay variation for corticosterone standards ranged from 2.5% to 5.7% and from 1.7% to 17% for extracted chicken pool plasma. Inter-assay variation of unextracted standard corticosterone was 7.2% and for extracted chicken pool plasma 21.3%.

### Immune function

To test the effect of traffic noise on immune function we measured leucocyte profiles of birds breeding in both treatments. We collected blood during the incubation period, at the same time as the samples for the corticosterone assays were collected, into separate capillaries and stored in separate Eppendorf tubes. Changes in leucocyte profiles, specifically, a higher heterophil/lymphocyte ratio (H/L ratio), have been found in several species of birds to be a good indicator of chronic stress (e.g. Vleck *et al*., 2000; Bedanova *et al*., 2010). We measured H/L ratios in birds twice, once when they were breeding in noise and once when they were breeding in the no-noise treatment and compared them within individuals.

We prepared blood smears using a standard two slide wedge procedure. The smears were air dried and then stained using the Differential Quik Stain Kit (Modified Giemsa). For each adult in the experiment we analysed two blood smears. In some preparations the smear was too thick and we were not able to see white blood cells, thus these samples were excluded, leaving a total sample size of 74 individuals. Each slide was inspected via a microscope with an oil immersion objective at ×100. We took a photo of every leucocyte that was present and counted the number of lymphocytes and granulocytes (eosinophils, basophils, heterophils) from the first 50 leucocytes we identified on each slide. We used the granulocyte to lymphocyte ratio as a proxy for heterophil to lymphocyte ratio (H/L ratio).

### Reproductive success

After a 2-month period during which the birds were allowed to habituate to the aviaries and began courtship behaviours, each aviary was supplied with 12 nest boxes and nesting materials (coconut fibers and cotton strings). Every other day, nests were checked and the fate of each egg and each offspring was recorded. Individuals attending the nests (that is, the social parents) were identified by observation in person or by video. Each social pair was allowed to produce only a single clutch in each treatment (i.e. once the first brood fledged, eggs from further breeding attempts were removed). When eggs hatched, nestlings were weighed, and at Day 8 we collected a blood sample (~10 μL) for parentage analysis (described below). When nestlings died before Day 8, we stored a tissue sample in ethanol for parentage analysis. In addition, we opened all eggs that did not hatch and scored the eggs as either infertile (i.e. no visible embryonic development) or as containing embryos that died before hatching. Tissue from dead embryos was collected and used for parentage analysis. As fitness measures for each bird we measured the proportion of genetic offspring that reached adulthood (120 days old) and the proportion of embryos that died before hatch (embryo mortality). In addition, every offspring was weighed at Days 10, 21 and 120 post-hatch.

### Paternity analysis

Since there is typically a considerable amount of extra-pair young in captive zebra finch colonies (Forstmeier *et al*., 2011), genetic paternity analysis is necessary to reliably assign parentage. To this end, all offspring were genotyped at 11 highly polymorphic microsatellite markers (chr2_109, chr2_47, chrZ_34, chr15_6, chr1a_39, chr22_3, chr3_58, chr11_8, chr27_1, chr5_34, chr6_16, see Table S2 in the online supplement for further information) following Forstmeier *et al*., (2007b) and Wang *et al*., (2017). Genetic parentage was determined by exclusion using the R package SOLOMON (Christie *et al*., 2013).

### Statistics

All statistical analyses were performed in R 3.1.1 (R Core Team, 2013). We applied linear mixed-effects and generalized mixed-effects models to analyse our data for which we used the ‘lmer’ and ‘glmer’ function from the lme4 package (Bates *et al*., 2015). Additionally, we used the ‘sim’ function from the arm package (Gelman and Hill, 2007) to simulate the posterior distribution of the model parameters based on 2000 simulations. The statistical significance of fixed effects was assessed based on the 95% credible intervals (CrIs) around the mean estimate (and 95% CrI are provided as number ranges in brackets following estimates). We considered an effect to be ‘significant’ in the frequentist’s sense when the 95% CrI did not overlap zero (Nakagawa and Cuthill, 2007). For approximate orientation we also calculated *P*-values from *t*-values or *z*-values assuming infinite degrees of freedom.

For ‘corticosterone baseline levels’ we used the ‘lmer’ function, and the corticosterone levels were ln-transformed to approach normality as the dependent variable. Treatment (0 = control, 1 = noise), sampling period (0 = pre-treatment, 1 = during courtship, 2 = during nesting/incubation or 3 = post-treatment), sex (0 = females, 1 = males) and breeding round (1 = first breeding round, 2 = breeding round) were set as independent factors and sampling time as covariate. Pre- and post-treatment sampling periods were coded as control for treatment, since during that time birds were not exposed to traffic noise. Sampling time refers to the moment the sample was taken counting from the moment we entered the room [during the first, second, third or fourth minute (the maximum time from entering the room to finishing the blood sample was 3.5 min)]. Every individual was exposed to control and noise treatment and blood samples were taken at four sampling times, thus individual ID was included as a random effect. Aviary was included as a second random effect to account for effects of the common aviary. In addition, we allowed for different slopes of individuals across the two treatments [random slope effect, since individuals had repeated measures under both treatments, usually 2× noise and 6× control, since in the noise treatment, the noise playback occurred only during the middle 2 of the 4 sampling times, the first and fourth corticosterone samples were coded as control].

For ‘immune response’, we used the ‘lmer’ function, ‘H/L ratio’ was log transformed to approach normality and was set as the dependent variable. Treatment (0 = control, 1 = noise) and sex (0 = females, 1 = males) were set as independent factors. The individual ID and the aviary (to account for effects of the common aviary) were included as random effects.

For our model of ‘reproductive success’, we used the ‘glmer’ function with a binomial error distribution. We assigned to every egg an ‘egg fate’ code, depending on the developmental stage they reached, and each individual was coded as 0 or 1 for each category: offspring reaching adulthood and embryo died before hatch (embryo mortality). We ran a model for each of those categories as the dependent variables (offspring reaching adulthood and embryo mortality). Treatment and breeding round were independent factors. The order of treatment refers to whether the birds were exposed to the noise treatment during their first or second round of breeding. In the models for offspring reaching adulthood and embryo mortality, genetic mother ID, genetic father ID, genetic pair ID and aviary were included as random effects. In some cases, some pairs switched partners between breeding rounds, thus genetic pair ID was included.

**Figure 2:**
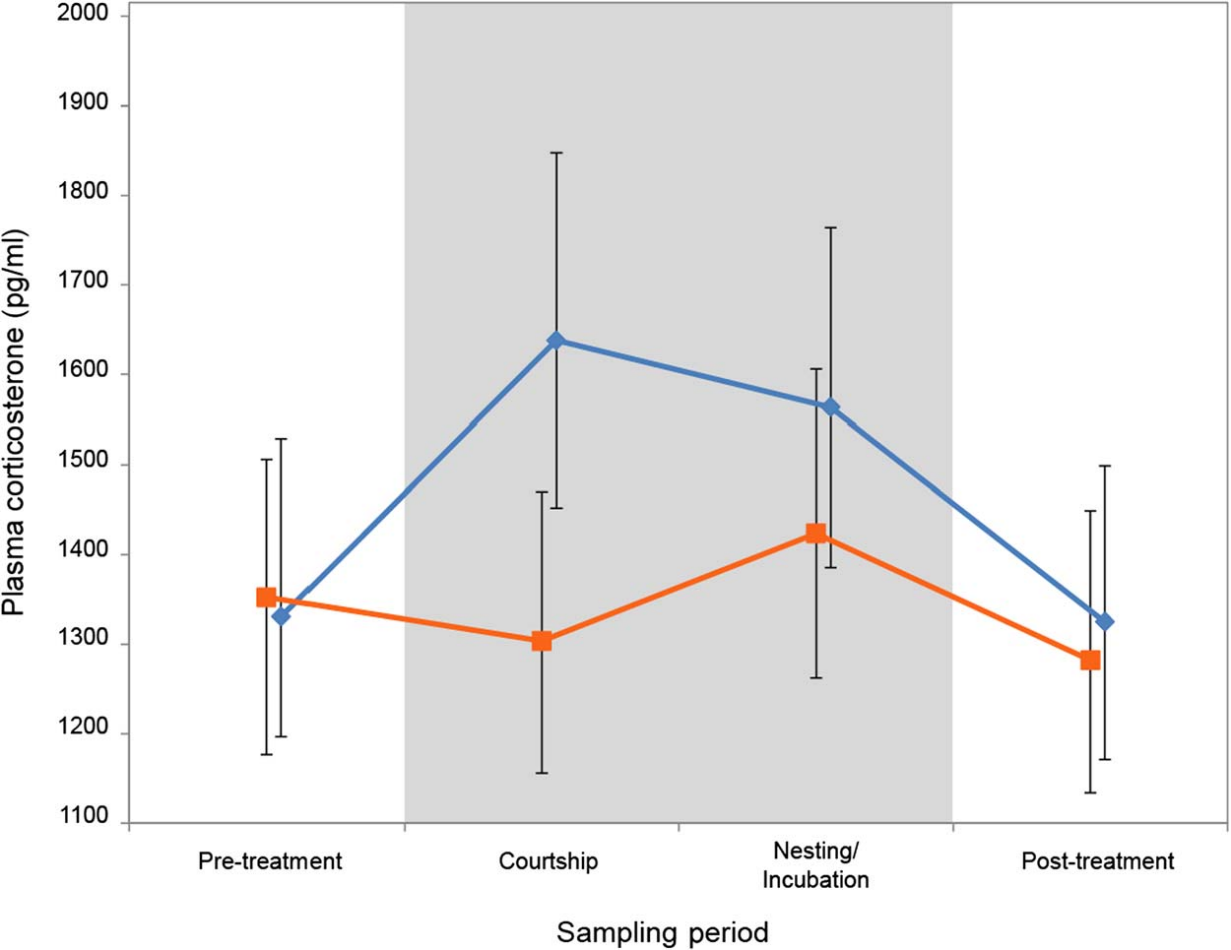
Mean baseline plasma corticosterone for noise exposed (orange squares) and control (no-noise playback, blue diamonds) breeding treatments at each sampling period. Error bars denote 95% CrIs.

The offspring body mass was measured three times per bird, when they were 10, 21 and 120 days old. We fitted three ‘lmer’ models, one model per age. As dependent variable we set mass, treatment and sex as independent factors. As random effects genetic mother ID, genetic father ID and aviary were included.

To measure extra-pair paternity each offspring was scored with 0 if its social father was the same as its genetic father and 1 otherwise. We fitted a ‘glmer’ model with a binomial error structure. As the dependent variable we set extra-pair paternity (yes/no) and as independent factors we set treatment, order of treatment (1 = control noise, 2 = noise control) and breeding round. Genetic mother ID, genetic clutch ID and aviary ID were included as random effects. We included the order of treatment since treatment could affect the establishment of couples, and therefore could affect extra-pair behaviour.

**Table 1:** Outcome of linear mixed-effects models testing the effects of noise on offspring mass at various ages, H/L ratio, plasma corticosterone levels, extra-pair paternity and reproductive success of adult zebra finches

**Mass Day 10**	**Random effects**	**Variance**	**Levels/Obs**				
	MotherID	0.70	39				
	FatherID	0	33				
	AviaryID	0.01	6				
	Residual	1.05					
	*N*		201				
	**Fixed effects**	**Estimate**	**Standard error (SE)**	***t***	***P***	**95% CI low**	**95% CI high**
	Intercept	10.64	0.192	55.41		10.254	11.011
	**Treatment** (Noise vs contr)	−0.39	0.159	−2.48	**0.013**	−0.708	−0.097
	Sex (male vs fem)	0.22	0.154	1.42	**0.156**	−0.084	0.518
**Mass Day 21**	**Random effects**	**Variance**	**Levels/Obs**				
	MotherID	0.07	39				
	FatherID	0.01	33				
	AviaryID	0.12	6				
	Residual	1.06					
	*N*		192				
	**Fixed effects**	**Estimate**	**SE**	***t***	***P***	**95% CI low**	**95% CI high**
	Intercept	12.22	0.199	61.31		11.830	12.624
	Treatment (noise vs contr)	−0.05	0.157	−0.33	**0.741**	−0.354	0.264
	Sex (male vs fem)	0.07	0.153	0.44	**0.660**	−0.217	0.370
**Mass Day 120**	**Random effects**	**Variance**	**Levels/Obs**				
	MotherID	0.05	39				
	FatherID	0.26	33				
	AviaryID	0.001	6				
	Residual	1.12					
	N		194				
	**Fixed effects**	**Estimate**	**SE**	***t***	***P***	**95% CI low**	**95% CI high**
	Intercept	14.80	0.171	86.72		14.481	15.152
	Treatment (noise vs contr)	−0.23	0.171	−1.36	**0.174**	−0.566	0.091
	Sex (male vs fem)	−0.15	0.162	−0.94	**0.347**	−0.483	0.170
**H/L ratio (log transf.)**	**Random effects**	**Variance**	**Levels/Obs**				
	IndividualID	0.07	74				
	AviaryID	0	6				
	Residual	0.69					
	N		144				
	**Fixed effects**	**Estimate**	**SE**	***t***	***P***	**95% CI low**	**95% CI high**
	Intercept	−0.33	0.125	−2.65		−0.579	−0.067
	Treatment (noise vs contr)	−0.22	0.139	−1.58	**0.114**	−0.492	0.055
	**Sex** (male vs fem)	−0.31	0.153	−2.04	**0.041**	−0.602	−0.018
**Corticosterone (log transf.)**	**Random effects**	**Variance**	**Levels/Obs**				
	IndividualID	0.06	87				
	Individual:treatment	0.06					
	AviaryID	0.06	6				
	Residual	0.34					
	N		629				
	**Fixed effects**	**Estimate**	**SE**	***t***	***P***	**95% CI low**	**95% CI high**
	Intercept	7.01	0.127	54.99		6.763	7.257
	**Treatment** (noise vs contr)	−0.16	0.069	−2.33	**0.019**	−0.294	−0.024
	**Period** (courtship vs pre)	0.16	0.074	2.24	**0.025**	0.017	0.313
	**Period** (nesting vs pre)	0.19	0.074	2.54	**0.011**	0.038	0.329
	Period (post vs pre)	−0.03	0.068	−0.43	**0.667**	−0.163	0.111
	**Sex** (male vs fem)	−0.16	0.068	−2.35	**0.019**	−0.296	−0.026
	**Breeding round** (2nd vs 1st)	−0.11	0.049	−2.35	**0.019**	−0.211	−0.019
	**Sampling time** (per min)	0.26	0.030	8.91	**<0.0001**	0.204	0.322
**Extra-pair paternity (binomial)**	**Random effects**	**Variance**	**Levels/Obs**				
	GeneticClutchID	11.79	61				
	MotherID	1.84	38				
	AviaryID	0	12				
	N		232				
	**Fixed effects**	**Estimate**	**SE**	***z***	***P***	**95% CI low**	**95% CI high**
	Intercept	0.45	1.148	0.389	**0.697**	−1.564	2.474
	Treatment (noise vs contr)	0.14	1.174	0.115	**0.908**	−1.969	2.177
	Order of treatment (noise first vs control first)	−2.47	1.378	−1.789	**0.074**	−4.706	−0.178
	Breeding round (2nd vs 1st)	−0.12	1.169	−0.102	**0.919**	−2.168	1.950
**Embryo mortality (binomial)**	**Random effects**	**Variance**	**Levels/Obs**				
	GeneticPairID	0.99	66				
	GeneticMotherID	1.03	40				
	GeneticFatherID	0.001	35				
	AviaryID	0.001	12				
	N		297				
	**Fixed effects**	**Estimate**	**SE**	***z***	***P***	**95% CI low**	**95% CI high**
	Intercept	−1.76	0.47	−3.708	**>0.001**	−2.654	−0.930
	Treatment (noise vs contr)	0.20	0.36	0.554	**0.579**	−0.467	0.872
	Order of treatment (noise first vs control first)	−0.33	0.56	−0.579	**0.563**	−1.373	0.740
	Breeding round (2nd vs 1st)	0.52	0.36	1.425	**0.154**	−0.169161	1.2209318
**Offspring survival (from early embryo to 120 days; binomial)**	**Random effects**	**Variance**	**Levels/Obs**				
	GeneticPairID	<0.001	66				
	GeneticMotherID	0.78	40				
	GeneticFatherID	<0.001	35				
	AviaryID	<0.001	12				
	N		297				
	**Fixed effects**	**Estimate**	**SE**	***z***	***P***	**95%CI low**	**95%CI high**
	Intercept	1.32	0.35	3.751	**<0.001**	0.583	1.998
	Treatment (noise vs contr)	−0.46	0.29	−1.559	**0.119**	−1.009	0.101
	Order of treatment (noise first vs control first)	0.59	0.41	1.441	**0.149**	−0.204	1.409
	**Breeding round** (2nd vs 1st)	−0.73	0.29	−2.483	**0.013**	−1.296	−0.162

## Results

### Baseline corticosterone levels

Baseline corticosterone levels were lower during the courtship period when birds were exposed to noise (Fig. 2). The baseline corticosterone levels did not differ from those of birds in the control treatment during the pre-treatment, and post-treatment sampling periods, when neither treatment group was exposed to noise. In addition, sex, breeding round and sampling period did not have a significant effect on corticosterone levels (Table 1). The estimate of individual repeatability for corticosterone levels was 0.121 (variance in random intercepts) and there was also variation in how individuals responded to the two treatments (0.116 variance in random slopes). It is worth noting that sampling time (the number of minutes after entering the aviary room that the blood sample was taken) had a highly significant effect on corticosterone levels (Table 1). This is contrary to the common belief that acute stress does not significantly impact baseline corticosterone levels if collected within 3 min of the onset of the disturbance (Romero and Reed, 2005). Our study, with an *N* of 629 samples, shows that plasma corticosterone does increase, highly significantly, from the first to the second minute post-disturbance, and by a factor of 2.36 times between the first and last category: from 950 to 2250 pg/ml. In our study, time (seconds after entering the room) explains about 11.6% of the variance in baseline corticosterone measures.

### Immune system

**Figure 3:**
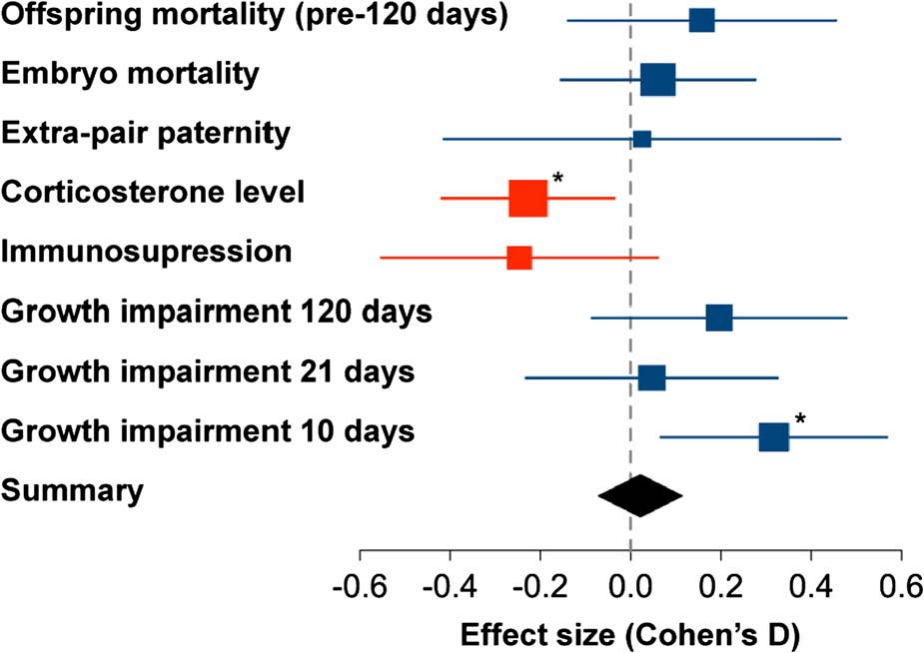
Effect size of each measured parameter for individuals in noise. Data points give the mean estimates of the models with 95% confidence intervals (1.96*SE). To make all effects comparable, variables were modelled as normally distributed, and all dependent variables were scaled to a standard deviation of 1 (scaled values can be found in Table S1, in the electronic supplemental materials). For illustration, the sign of the effect of noise was standardized such that we expected to obtain values >0 (e.g. the effect on offspring mass was multiplied by −1 in order to reflect growth impairment rather than growth). Data point size reflects the sample size. Red symbols indicate effects that were opposite to expectations. Asterisks indicate significance, without correction for multiple testing.

Traffic noise did not have a significant effect on granulocyte/lymphocyte (H/L) ratio in adult zebra finches (Table 1). Birds exposed to noise showed a non-significant tendency towards lower H/L ratios (Fig. 3), opposite to the predicted direction—birds in noise exposure treatment had an estimated ratio of 0.68 (95% CrI 0.52–0.82) and control birds had a ratio of 0.87 (0.72–1.03) H/L (parameter estimates calculated on data prior to ln-transformation, hence different to the models in Table 1). Males had significantly lower H/L ratios than females [males 0.66 (0.50–0.81) H/L, females 0.89 (0.74–1.03) H/L].

### Reproductive success and extra-pair paternity

The traffic noise treatment did not have a significant effect on the proportion of fertile eggs that reached adulthood (Table 1), although there was a trend towards higher offspring mortality (Fig. 3). Birds had a significantly lower proportion of offspring surviving to adulthood in the second breeding round (Table 1), regardless of noise treatment.

Overall embryo mortality was 24% (*N* = 296), but we did not find significant differences in embryo mortality levels between noise and control treatments (Table 1).

Likewise, we found an overall level of extra-pair paternity of 42%, but the proportion of extra-pair paternity was not affected by traffic noise, breeding round or order of treatment (Table 1).

The offspring of parents in the noise exposure treatment were significantly lighter at 10 days post-shatch than those born to the same parents in control treatments (Table 1, effect estimate 0.39 g), but did not differ in body mass at later stages, although there were trends towards smaller mass for chicks born to noise-exposed parents at all three age categories (Fig. 3). The estimate of body mass of the offspring at Day 10 was 10.36 g (10.01–10.72) in the noise treatment and 10.75 g (10.41–11.08) in the control treatment; at 21 days 12.21 g (11.85–12.59) in noise and 12.26 g (11.90–12.61) in controls and at 120 days 14.49 g (14.17–14.80) in noise 14.72 g (14.43–15.02) in controls. No significant difference in body mass between female and male offspring could be detected (Table 1).

## Discussion

We found that traffic noise had a small but significant effect on baseline corticosterone levels, which were lower in noise-exposed birds in the courtship, nest building, egg laying or incubation period of the reproductive cycle. These differences did not continue after the noise treatment ended, when their offspring reached adulthood (120 days post-hatch). We also found that offspring raised in noise conditions were smaller at 10 days post-hatch than offspring of the same parents raised in no-noise conditions. In our experiment, chronic traffic noise exposure did not have a direct, significant impact on any of the other variables we measured, although birds in noise treatments tended to have lower H/L ratios (a trend opposite to our prediction) and higher levels of offspring mortality than birds in a no-noise treatment.

### Baseline corticosterone levels

We found that baseline corticosterone levels increased during the courtship and breeding periods in control birds (Fig. 2). However, when the same birds were chronically exposed to traffic noise during the same part of the reproductive cycle, their baseline corticosterone levels remained low. To date, previous studies investigating links between noise exposure and glucocorticoid stress responses have produced very mixed evidence. Some studies reported positive correlations between noise exposure and baseline corticosterone levels in adult songbirds (Davies *et al*., 2017), while others found no change in corticosterone baselines (Potvin and MacDougall-Shackleton, 2015a; Injaian *et al*., 2018), and still others found lower baseline corticosterone in noise-exposed adults (Kleist *et al*., 2018). These differences could result from methodological differences, as the noise exposure in these studies varied in both their duration (3 min to chronic throughout the lifetime of the bird) and in their composition (broad spectrum and constant amplitude to varying in both frequency content and amplitude over time), or they could be specific to a certain species (Kleist *et al*., 2018), their ontogenetic background or experience (Davies *et al*., 2017) or to geographic location (Partecke, 2013). Note that in our study, the observed effect is of small magnitude (see Fig. 2), reaching statistical significance based on >600 measures of corticosterone. Such effects would not be detectable with the usual sample sizes, which are often more than an order of magnitude smaller.

In animals exposed to a chronic stressor, it has been found in some cases that baseline levels of corticosterone are higher than in non-stressed individuals, but in other cases, they may have lower baselines corticosterone than controls. This is because animals might deal with long-term disturbances by suppressing corticosteroid responses (Rich and Romero, 2005). Birds may physiologically reduce activity of the hypothalamic–pituitary–adrenal (HPA) axis during periods of chronic stress to elude pathological effects linked with chronically elevated glucocorticoid concentrations, such as weight loss, impaired immune function and hyperglycemia (Cyr and Romero, 2007, 2009). Our birds were found to have lower levels of baseline corticosterone when breeding in traffic noise than they did during the no noise treatment, particularly at the second date, which occurred when the birds were in the pair formation and courtship period of the reproductive cycle. This difference does not represent a decrease from the pre-treatment baseline, but rather results from a lack of an increase in baseline corticosterone that was observed in the control condition. One hypothesis for this apparent suppression of the elevated corticosterone baseline during reproduction could be that chronic noise reduces corticosterone responsiveness in order to minimize the negative impacts of chronically elevated glucocorticoids as found by Cyr and Romero (2007, 2009). While we did not find differences in immune function in our noise exposed birds, without additional tests we cannot confirm that our birds in noise had lower corticosterone during breeding than controls because of a suppression of the normal glucocorticoid response or for some other reason.

While we did not find differences between treatments in H/L ratios, a measure of immune function, nor in our measures of reproductive success, we cannot yet conclude that our birds were habituated to the traffic noise. To test whether birds in chronic noise are down-regulating the activity of the HPA axis rather than simply becoming habituated to the noise, it would be necessary to follow up with further experimental testing, such as measuring acute glucocorticoid responses to a second, novel stressor (Wingfield and Romero, 2015).

Another possible interpretation of our results could be that traffic noise is initially a potent stressor, but that the birds quickly habituate to the noise (i.e. the birds learn that the stimulus is not harmful or aversive). To rule out habituation, one must look beyond baseline glucocorticoid levels at other measures, such as immune function, behavioural changes, oxidative damage on the cellular level or secondary responses to additional stressors (Wingfield and Romero, 2015). For instance, European starlings exposed to chronic stress had lower corticosterone levels than controls, but also had reduced reproductive success (Cyr and Romero, 2007), suffered decreases in body weight and altered blood chemistry (Awerman and Romero, 2010) and showed depressed corticosterone responses to novel stressors (Rich and Romero, 2005), all of which are diagnostic indications that habituation had not occurred (Cyr and Romero, 2009). In this study we investigated not only plasma corticosterone but also several measures of reproductive success, extra-pair paternity and immune function, in order to have a wider perspective on potential physiological consequences of chronic noise exposure in zebra finches.

### Immune system

Zebra finches exposed to chronic noise did not have significantly higher H/L ratios, as we had predicted. Previous studies have found that an increase in H/L ratio was a reliable indicator of chronic stress in birds, including songbirds (Fourie and Hattingh, 1983; Gross and Siegel, 1986; Maxwell, 1993; Campbell, 1995; Ots and Horak, 1996; Müller *et al*., 2011). One study in chickens even found a noise-related increase in H/L ratio when chronically exposing them to a very loud unfamiliar sound (Bedanova *et al*., 2010). However, our study showed an opposite tendency to what we expected, namely a non-significant reduction in the H/L ratio for individuals exposed to noise. It is possible that we did not observe differences in H/L ratios, not because of a lack of increase in heterophils, but rather because there was also an increase in lymphocytes. An increase of lymphocytes could be related with the mating, nesting, hatching and chick rearing periods if birds need to be in the better condition to resist this demanding process. As we only measured H/L ratio at one point in the reproductive cycle, we may have missed an effect if one occurred earlier or later in the treatment. Also, it is important to take into account that other environmental conditions were stable in our study, such as ad lib food availability. Thus, birds might have, for instance, limited negative effects of other stressors on their immune system by changing food intake (Klasing, 2007). Finally, our data did show a differential response in females and males, in that males had lower H/L ratios. This observation suggests that breeding behaviour and/or being in reproductive condition may have different physiological consequences for males than for females.

### Reproductive success and extra-pair paternity

As measures of reproductive success, we measured the proportion of offspring that reached adulthood and embryo mortality (embryos that died before hatching). We did not find any significant correlations between noise and either of these variables. However, offspring mortality tended to be higher in birds exposed to noise. In addition to these measures of reproductive success, we also measured body mass in chicks. We measured the body mass of offspring at 10, 21 and 120 days of age and we found that the offspring in the noise treatment were slightly, but significantly lighter at 10 days of age than those in the control treatment. However, the offspring from the two treatments were not different in mass by age 21 days, and so it is unclear whether the small difference in mass during the nestling phase would have much impact on the condition or fitness of these offspring later in life.

Several field studies reported correlations between anthropogenic noise and reproductive success (Kuitunen *et al*., 2003; Halfwerk *et al*., 2011; Kight *et al*., 2012; Injaian *et al*., 2018; Kleist *et al*., 2018). Western bluebirds (*Sialia mexicana*) near noisy oil and gas extraction sites had lower hatching success than conspecifics living near similar, but silent, oil and gas well pads (Kleist *et al*., 2018). European pied flycatchers (*Ficedula hypoleuca*) had a decreased number of fledglings per breeding attempt when they lived close to roads (Kuitunen *et al*., 2003). Both great tits and eastern bluebirds nesting in areas with higher levels of anthropogenic noise fledged fewer offspring than conspecifics in quieter sites (Halfwerk *et al*., 2011; Kight *et al*., 2012). Additionally, the offspring of tree swallows (*Tachycineta bicolor*) exposed to noise during breeding were smaller, fledged later and experienced higher levels of oxidative stress and had a higher rate of telomere attrition than those in quieter sites (Injaian *et al*., 2018, 2019). While we did not find a striking effect of experimental chronic traffic noise exposure on reproductive success in our laboratory experiment, we did find a tendency towards higher offspring mortality and a small but significant effect of lower body mass in chicks from parents exposed to noise. Our results may be accounted for by direct noise effects on the offspring embryos and hatchlings or by indirect effects via modulation of parental behaviours such as feeding rate. Supporting the latter idea, feeding rates in other songbird species have been shown to be lower in birds raising their young in noisy environments, possibly as a result of noise masking the begging calls of young in the nest (Leonard and Horn, 2008; Schroeder *et al*., 2012; Leonard *et al*., 2015). Begging calls are essential in parent–offspring communication since chicks produce begging calls that elicit feeding behaviour in adults (Burford *et al*., 1998; Budden and Wright, 2001). However, it will require experiments specifically targeting this issue in the future to work out exactly if and how noise exposure may influence parental feeding behaviour and how that affects reproductive success in zebra finches.

In line with our observation that offspring of birds exposed to noise were lighter at the nestling stage (10 days post-hatch) than the offspring from birds in quiet aviaries, previous studies have found that noise during ontogeny can retard growth, particularly in young chicks that are still in the nest (Potvin and MacDougall-Shackleton, 2015a). For example, noise exposure during egg development resulted in chicks with lower body and brain mass in domestic chickens *Gallus gallus* (Kesar, 2014). Chronic noise exposure impairs normal brain development in the auditory cortex in rats (Chang, 2003) and in areas of the song control system in zebra finches (Potvin *et al*., 2016). Further, in a related study, we found that juvenile zebra finches exposed to the same noise treatments as we used in this study had higher rates of telomere loss than juveniles from control groups or juveniles whose parents were exposed to noise (Dorado-Correa *et al*., 2018). Together, this evidence indicates that anthropogenic noise may indeed be an important source of stress-related health effects and is particularly potent during early life.

While our study did not find statistically significant effects of noise playback during breeding on the immune system and reproductive success of the parents, it may be premature to conclude that chronic noise has no negative impact on urban birds. Our experimental birds were housed in climate controlled, parasite- and predator-free aviaries, with unlimited access to food and water. They were not exposed to chemical pollutants, nor to light pollution at night. It may be that noise in itself is indeed a stressor, but not potent enough to cause serious effects on the breeding success or health of adult birds. In humans, noise effects on health may be augmented by, or in turn, may increase the impact of other stressors (reviewed in Stansfeld and Matheson, 2003). Similar results to those we report here have been found in response to other types of stressors in zebra finches breeding in captivity. For example, when breeding pairs were required to spend more time foraging for food, their offspring were lighter at fledging than offspring from parents with easy access to food (Spencer *et al*., 2003; Brumm *et al*., 2009; Honarmand *et al*., 2017). However, it is feasible to imagine that traffic noise pushes wild birds, which already experience a range of environmental challenges and stressors, closer to a threshold above which the combined effects of these stressors would result in more severe physiological effects and fitness consequences. To truly understand the effects of urban environments on bird fitness, it will thus be necessary to conduct experiments designed to investigate the combined effects of multiple aspects of urbanization.

## Conclusion

This study is one of the first comprehensive experimental tests of effects of traffic noise exposure on captive songbirds, investigating reproductive success, immune function, glucocorticoid levels and extra-pair paternity in a repeated measures design. We found that noise depressed corticosterone levels in breeding birds and reduced the growth of their nestlings, but that noise did not, by itself, induce acute negative effects on immune status, reproductive success or extra-pair paternity in exposed breeding adults. Further studies into whether noise exposure may have more long-term fitness-related consequences, and if noise interacting with additional stressors leads to more serious acute effects, are needed before ruling out noise as a potent threat to the health and fitness of exposed wildlife. The results of our study correspond with the findings of a recent noise study (Kleist *et al*., 2018), but not with others (Potvin and MacDougall-Shackleton, 2015a; Davies *et al*., 2017; Injaian *et al*., 2018) in the details of when and in which way noise affects reproductive success and physiology. These differences make clear the importance of taking life history stage, species traits and noise profiles into account when designing future experiments, as one-off or short-term studies could easily underestimate impacts of noise pollution. Our study further adds to existing evidence that noise has a stronger negative impact on adult birds during more vulnerable life history stages, such as reproduction, nestling care, as well as during early ontogeny. In the face of increasing urbanization globally, it is critical to consider how to create urban environments that are as ‘livable’ for as many species as possible. Given that global transportation infrastructure spending and car ownership is predicted to double in the next 10 years (Oxford Economics, 2017), traffic noise exposure will potentially affect more wildlife than ever before. Noise mitigation strategies such as sinking roads below ground level or constructing walls to insulate surrounding areas from noise (Nemeth and Zollinger, 2014), creating quieter road surfaces (e.g. Kleizienė et al., 2019) and quieter vehicles can protect sensitive species from some of the negative impacts of road noise year round. However, our study suggests that when permanent noise-reducing structures are not possible, noise impacts could be lessened by developing more transient noise-mitigating strategies, for example, which are focused on reducing road noise during seasonal peaks in avian reproductive behaviour.

## Supplementary Material

Zollinger_et_al_Supplement_coz056
