# Supplementary material for: Traffic noise exposure depresses plasma corticosterone and delays offspring growth in breeding zebra finches
Source: Conserv Physiol. 2019 Oct 11;7(1):coz056. doi: 10.1093/conphys/coz056 (PMC6788579; doi:10.1093/conphys/coz056)
Supplement: Zollinger_et_al_Supplement_coz056 [file zollinger_et_al_supplement_coz056.docx]

Electronic Supplement

# Traffic noise exposure depresses plasma corticosterone and delays offspring growth in breeding zebra finches

Sue Anne Zollinger^1,2*^, Adriana Dorado-Correa^2^, Wolfgang Goymann^3^, Wolfgang Forstmeier^4^, Ulrich Knief^4,5^, Ana Maria Bastidas^6^, Henrik Brumm^2^

^1^Department of Natural Sciences, Manchester Metropolitan University, Manchester M1 5GD United Kingdom. [zollinger@orn.mpg.de](mailto:zollinger@orn.mpg.de)

^2^Communication and Social Behaviour Group and ^3^Department of Behavioural Neurobiology and

^4^Department of Behavioural Ecology and Evolutionary Genetics, Max Planck Institute for Ornithology, 82319 Seewiesen, Germany

^5^Evolutionary and Ecological Genetics Group, and ^6^Munich Graduate School for Evolution, Ecology and Systematics, Ludwig Maximillians University, 82157 Munich, Germany

Table S1: Outcome of linear mixed-effects models with a Gaussian error structure testing the effects of noise on corticosterone leves, H/L ratio, reproductive success and extra-pair paternity of adult zebra finches exposed to traffic noise or a no-noise control treatment. The dependent variables were Z-transformed according to Gelman and Hill (2007), and these scaled values were used in the estimations of effect sizes presented in the forest plot summary graph, Figure 3.

| Dependent Var | Random effects | Variance | Levels/Obs |  |  |  |  |
| --- | --- | --- | --- | --- | --- | --- | --- |
| **Mass day 10** | **MotherID** | **0.70** | **39** |  |  |  |  |
|  | FatherID | 0.00 | 33 |  |  |  |  |
|  | AviaryID | 0.01 | 6 |  |  |  |  |
|  | Residual | 1.05 |  |  |  |  |  |
|  | N |  | 201 |  |  |  |  |
|  | Fixed effects | Estimate | SE | t | p | 95%CI low | 95%CI high |
|  | Intercept | 10.64 | 0.192 | 55.41 |  | 10.254 | 11.011 |
|  | Treatment (noise vs contr) | -0.39 | 0.159 | -2.48 | 0.013 | -0.709 | -0.097 |
|  | Sex (male vs fem) | 0.22 | 0.154 | 1.42 | 0.156 | -0.084 | 0.518 |
|  |  |  |  |  |  |  |  |
| **Mass day 21** | **Random effects** | **Variance** | **Levels/Obs** |  |  |  |  |
|  | MotherID | 0.07 | 39 |  |  |  |  |
|  | FatherID | 0.01 | 33 |  |  |  |  |
|  | AviaryID | 0.12 | 6 |  |  |  |  |
|  | Residual | 1.06 |  |  |  |  |  |
|  | N |  | 192 |  |  |  |  |
|  | Fixed effects | Estimate | SE | t | p | 95%CI low | 95%CI high |
|  | Intercept | 12.23 | 0.199 | 61.31 |  | 11.830 | 12.625 |
|  | Treatment (noise vs contr) | -0.05 | 0.157 | -0.33 | 0.741 | -0.354 | 0.264 |
|  | Sex (male vs fem) | 0.07 | 0.154 | 0.44 | 0.660 | -0.217 | 0.370 |
|  |  |  |  |  |  |  |  |
| **Mass day 120** | **Random effects** | **Variance** | **Levels/Obs** |  |  |  |  |
|  | MotherID | 0.05 | 39 |  |  |  |  |
|  | FatherID | 0.26 | 33 |  |  |  |  |
|  | AviaryID | 0.00 | 6 |  |  |  |  |
|  | Residual | 1.12 |  |  |  |  |  |
|  | N |  | 194 |  |  |  |  |
|  | Fixed effects | Estimate | SE | t | p | 95%CI low | 95%CI high |
|  | Intercept | 14.80 | 0.171 | 86.72 |  | 14.481 | 15.152 |
|  | Treatment (noise vs contr) | -0.23 | 0.171 | -1.36 | 0.174 | -0.566 | 0.091 |
|  | Sex (male vs fem) | -0.15 | 0.162 | -0.94 | 0.347 | -0.483 | 0.170 |
|  |  |  |  |  |  |  |  |
| **H/L ratio (ln transf)** | **Random effects** | **Variance** | **Levels/Obs** |  |  |  |  |
|  | IndividualID | 0.07 | 74 |  |  |  |  |
|  | AviaryID | 0.00 | 6 |  |  |  |  |
|  | Residual | 0.69 |  |  |  |  |  |
|  | N |  | 144 |  |  |  |  |
|  | Fixed effects | Estimate | SE | t | p | 95%CI low | 95%CI high |
|  | Intercept | -0.33 | 0.125 | -2.65 |  | -0.579 | -0.067 |
|  | Treatment (noise vs contr) | -0.22 | 0.139 | -1.58 | 0.114 | -0.492 | 0.055 |
|  | Sex (male vs fem) | -0.31 | 0.153 | -2.04 | 0.041 | -0.602 | -0.018 |
|  |  |  |  |  |  |  |  |
| **Cort (ln transf)** | **Random effects** | **Variance** | **Levels/Obs** |  |  |  |  |
|  | IndividualID | 0.06 | 87 |  |  |  |  |
|  | Individual:treatment | 0.06 |  |  |  |  |  |
|  | AviaryID | 0.06 | 6 |  |  |  |  |
|  | Residual | 0.34 |  |  |  |  |  |
|  | N |  | 629 |  |  |  |  |
|  | Fixed effects | Estimate | SE | t | p | 95%CI low | 95%CI high |
|  | Intercept | 7.01 | 0.128 | 54.99 |  | 6.764 | 7.257 |
|  | Treatment (noise vs contr) | -0.16 | 0.069 | -2.33 | 0.020 | -0.294 | -0.024 |
|  | Period (courtship vs pre) | 0.17 | 0.074 | 2.24 | 0.025 | 0.017 | 0.313 |
|  | Period (nesting vs pre) | 0.19 | 0.074 | 2.54 | 0.011 | 0.038 | 0.329 |
|  | Period (post vs pre) | -0.03 | 0.068 | -0.43 | 0.667 | -0.163 | 0.111 |
|  | Sex (male vs fem) | -0.16 | 0.068 | -2.35 | 0.019 | -0.296 | -0.026 |
|  | Breeding round (2nd vs 1st) | -0.11 | 0.049 | -2.35 | 0.019 | -0.211 | -0.019 |
|  | Sampling order (per min) | 0.26 | 0.029 | 8.91 | <0.0001 | 0.204 | 0.322 |
|  |  |  |  |  |  |  |  |
| **EPP (binomial)** | **Random effects** | **Variance** | **Levels/Obs** |  |  |  |  |
| (42% EPP) | GeneticClutchID | 11.79 | 61 |  |  |  |  |
|  | MotherID | 1.84 | 38 |  |  |  |  |
|  | AviaryID | 0.00 | 12 |  |  |  |  |
|  | N |  | 232 |  |  |  |  |
|  | Fixed effects | Estimate | SE | z | p | 95%CI low | 95%CI high |
|  | Intercept | 0.45 | 1.148 | 0.38 | 0.697 | -1.567 | 2.474 |
|  | Treatment (noise vs contr) | 0.14 | 1.174 | 0.11 | 0.908 | -1.969 | 2.177 |
|  | Order of treatment (noise first vs control first) | -2.47 | 1.378 | -1.79 | 0.074 | -4.706 | -0.178 |
|  | Breeding round (2nd vs 1st) | -0.12 | 1.169 | -0.10 | 0.919 | -2.168 | 1.950 |
|  |  |  |  |  |  |  |  |
| **Embryo mortality (binomial)** | **Random effects** | **Variance** | **Levels/Obs** |  |  |  |  |
| (24% EM) | GeneticPairID | 0.99 | 66 |  |  |  |  |
|  | GeneticMotherID | 1.03 | 40 |  |  |  |  |
|  | GeneticFatherID | 0.00 | 35 |  |  |  |  |
|  | AviaryID | 0.00 | 12 |  |  |  |  |
|  | N |  | 297 |  |  |  |  |
|  | Fixed effects | Estimate | SE | z | p | 95%CI low | 95%CI high |
|  | Intercept | -1.76 | 0.474 | -3.71 | 0.000 | -2.654 | -0.930 |
|  | Treatment (noise vs contr) | 0.20 | 0.363 | 0.55 | 0.579 | -0.467 | 0.872 |
|  | Order of treatment (noise first vs control first) | -0.33 | 0.566 | -0.58 | 0.563 | -1.373 | 0.740 |
|  | Breeding round (2nd vs 1st) | 0.52 | 0.363 | 1.42 | 0.154 | -0.169 | 1.221 |
|  |  |  |  |  |  |  |  |
| **Offspring survival (from early embryo to 120days; binomial)** | **Random effects** | **Variance** | **Levels/Obs** |  |  |  |  |
| (69% survival) | GeneticPairID | 0.00 | 66 |  |  |  |  |
|  | GeneticMotherID | 0.78 | 40 |  |  |  |  |
|  | GeneticFatherID | 0.00 | 35 |  |  |  |  |
|  | AviaryID | 0.00 | 12 |  |  |  |  |
|  | N |  | 297 |  |  |  |  |
|  | Fixed effects | Estimate | SE | z | p | 95%CI low | 95%CI high |
|  | Intercept | 1.32 | 0.353 | 3.75 | 0.000 | 0.583 | 1.998 |
|  | Treatment (noise vs contr) | -0.46 | 0.294 | -1.56 | 0.119 | -1.009 | 0.100 |
|  | Order of treatment (noise first vs control first) | 0.59 | 0.410 | 1.44 | 0.149 | -0.204 | 1.409 |
|  | Breeding round (2nd vs 1st) | -0.73 | 0.294 | -2.48 | 0.013 | -1.296 | -0.162 |

**Table S2.** Microsatellite markers used in the parentage genotyping analysis.

| **chromosome** | **microsatellite** | **start** | **end** | **Microsatellite**  **position** | **primer name** | **Tm (MWG) [°C]** | **sequence** |
| --- | --- | --- | --- | --- | --- | --- | --- |
| *Tgu24* | chr24_2 | 2,054,918 | 2,054,951 | [chr24:2054918-2054951](http://genome.ucsc.edu/cgi-bin/hgTracks?hgsid=210650717&db=taeGut1&position=chr24%3A2054918-2054951) | -F | 59.83 (59.35) | AGCCGAGGAAGGTGGATGTT |
|  |  |  |  |  | -R | 60.3 (59.35) | GGACCCAGCTTTGCCTTGAT |
| *Tgu2* | chr2_47 | 46,750,591 | 46,750,670 | [chr2:46750591-46750670](http://genome.ucsc.edu/cgi-bin/hgTracks?hgsid=210650717&db=taeGut1&position=chr2%3A46750591-46750670) | -F | 59.29 (57.30) | TCCCAAACAGCACTTCCACA |
|  |  |  |  |  | -R | 58.08 (57.30) | TTTGCCCTTACACCACCTGA |
| *Tgu2* | chr2_109 | 109,436,800 | 109,436,848 | [chr2:109436800-109436848](http://genome.ucsc.edu/cgi-bin/hgTracks?hgsid=210650717&db=taeGut1&position=chr2%3A109436800-109436848) | -F | 59.55 (57.30) | TCGTTTGTGCCTCTCGATCA |
|  |  |  |  |  | -R | 58.41 (57.30) | TGTTGCCCTCCAGTCTGAAA |
| *TguZ* | chrZ_34 | 34,494,607 | 34,494,681 | [chrZ:34494607-34494681](http://genome.ucsc.edu/cgi-bin/hgTracks?hgsid=210650717&db=taeGut1&position=chrZ%3A34494607-34494681) | -F | 60.14 (59.35) | CCCTGACACAAAGGCTGCAT |
|  |  |  |  |  | -R | 58.38 (59.35) | GCTCTCAGCCTTGCTGCATA |
| *Tgu15* | chr15_6 | 6,303,384 | 6,303,431 | [chr15:9322101-9322138](http://genome.ucsc.edu/cgi-bin/hgTracks?hgsid=211552843&db=taeGut1&position=chr15%3A9322101-9322138) | -F | 59.93 (59.35) | AGCCGAGGGCCTAAAGATGA |
|  |  |  |  |  | -R | 57.21 (59.35) | GAGCCAGGATGAAAGGAGGT |
| *Tgu1A* | chr1A_39 | 30,057,561 | 30,057,609 | [chr1A:30057561-30057609](http://genome.ucsc.edu/cgi-bin/hgTracks?hgsid=210650717&db=taeGut1&position=chr1A%3A30057561-30057609) | -F | 60.22 (61.78) | GGCTCCTTAAAAGCCCAGCTC |
|  |  |  |  |  | -R | 53.40 (61.40) | CTCTGCTGGACCCTCTCTAG |
| *Tgu22* | chr22_3 | 2,509,679 | 2,509,714 | [chr22:2509679-2509714](http://genome.ucsc.edu/cgi-bin/hgTracks?hgsid=210650717&db=taeGut1&position=chr22%3A2509679-2509714) | -F | 60.15 (59.35) | TGGCCTTGCTGACTTCTGCT |
|  |  |  |  |  | -R | 58.82 (59.35) | AGCAGGTTGTGAGGGCTTGT |
| *Tgu3* | chr3_58 | 58,288,041 | 58,288,084 | [chr3:58288041-58288084](http://genome.ucsc.edu/cgi-bin/hgTracks?hgsid=210650717&db=taeGut1&position=chr3%3A58288041-58288084) | -F | 59.87 (59.35) | CCTGATTCACCATGCCCAGT |
|  |  |  |  |  | -R | 59.90 (60.65) | AAAGGGCAGAAGGTAGACCATGA |
| *Tgu11* | chr11_8 | 8,246,464 | 8,246,538 | [chr11:8246464-8246538](http://genome.ucsc.edu/cgi-bin/hgTracks?hgsid=210650717&db=taeGut1&position=chr11%3A8246464-8246538) | -F | 60.09 (59.35) | TTGCAGGCAGGTTCAGTGTG |
|  |  |  |  |  | -R | 60.10 (59.35) | TGGTTGCCTGGAGAAGATGG |
| *Tgu27* | chr27_1 | 1,136,423 | 1,136,454 | [chr27:1136423-1136454](http://genome.ucsc.edu/cgi-bin/hgTracks?hgsid=210650717&db=taeGut1&position=chr27%3A1136423-1136454) | -F | 56.52 (59.82) | GATCTGGAAATACCCTGGAGC |
|  |  |  |  |  | -R | 59.48 (60.25) | TGAAGCATTTCCCTCTGGAGTC |
| *Tgu5* | chr5_34 | 34,270,805 | 34,270,919 | [chr5:34270805-34270919](http://genome.ucsc.edu/cgi-bin/hgTracks?hgsid=210650717&db=taeGut1&position=chr5%3A34270805-34270919) | -F | 58.46 (59.35) | GCAACTGCTGCTCTGAAGGA |
|  |  |  |  |  | -R | 59.78 (59.35) | AGCTGCACATGGGGAAGCTA |
| *Tgu6* | chr6_16 | 16,264,863 | 16,264,907 | [chr6:16264863-16264907](http://genome.ucsc.edu/cgi-bin/hgTracks?hgsid=210650717&db=taeGut1&position=chr6%3A16264863-16264907) | -F | 59.95 (59.35) | TCTGCCGTGTGTGTTTCTGG |
|  |  |  |  |  | -R | 59.74 (59.35) | TAGCCATCTGGGCTCCTCAA |

**R Scripts used in analyses**

rm(list=ls())

library(lme4)

library(arm)

# (1) Body mass ----------------------------------------------------------------------------------

dat <- read.table(paste(path,"data_mass.txt",sep=""), sep="\t", header=TRUE)

dat10 <- subset(dat, day==10)

dat21 <- subset(dat, day==21)

dat120 <- subset(dat, day==120)

mod1 <- lmer(scale(mass) ~ factor(treat) + factor(sex) + (1|MoID) + (1|FaID) + (1|aviary), data=dat10)

summary(mod1)

sim.mod1 <- fixef(sim(mod1, n=2000))

quantile(sim.mod1[,1],c(0.025,0.975))

quantile(sim.mod1[,2],c(0.025,0.975))

quantile(sim.mod1[,3],c(0.025,0.975))

mod2 <- lmer(scale(mass) ~ factor(treat) + factor(sex) + (1|MoID) + (1|FaID) + (1|aviary), data=dat21)

summary(mod2)

sim.mod2 <- fixef(sim(mod2, n=2000))

quantile(sim.mod2[,1],c(0.025,0.975))

quantile(sim.mod2[,2],c(0.025,0.975))

quantile(sim.mod2[,3],c(0.025,0.975))

mod3 <- lmer(scale(mass) ~ factor(treat) + factor(sex) + (1|MoID) + (1|FaID) + (1|aviary), data=dat120)

summary(mod3)

sim.mod3 <- fixef(sim(mod3, n=2000))

quantile(sim.mod3[,1],c(0.025,0.975))

quantile(sim.mod3[,2],c(0.025,0.975))

quantile(sim.mod3[,3],c(0.025,0.975))

# (2) H/L ----------------------------------------------------------------------------------------

dat <- read.table(paste(path,"data_hlratio.txt",sep=""), sep="\t", header=TRUE)

dat$LOGratio <- log(dat$ratio)

mod4 <- lmer(scale(LOGratio) ~ factor(treat) + factor(sex) + (1|ID) + (1|Aviary), data=dat)

summary(mod4)

sim.mod4 <- fixef(sim(mod4, n=2000))

quantile(sim.mod4[,1],c(0.025,0.975))

quantile(sim.mod4[,2],c(0.025,0.975))

quantile(sim.mod4[,3],c(0.025,0.975))

# (3) Cort ---------------------------------------------------------------------------------------

dat <- read.table(paste(path,"data_cort.txt",sep=""), sep="\t", header=TRUE)

dat$LOGcort <- log(dat$cort)

is.numeric(dat$Order)

mod5 <- lmer(scale(LOGcort) ~ factor(treat) + factor(Time) + factor(sex) + factor(Round) + Order + (1|Aviary) + (treat|ID), data=dat)

summary(mod5) # --> very different

sim.mod5 <- fixef(sim(mod5, n=2000))

quantile(sim.mod5[,1],c(0.025,0.975))

quantile(sim.mod5[,2],c(0.025,0.975))

quantile(sim.mod5[,3],c(0.025,0.975))

quantile(sim.mod5[,4],c(0.025,0.975))

quantile(sim.mod5[,5],c(0.025,0.975))

quantile(sim.mod5[,6],c(0.025,0.975))

quantile(sim.mod5[,7],c(0.025,0.975))

quantile(sim.mod5[,8],c(0.025,0.975))

# for Fig

mod5fig <- lmer(LOGcort ~ -1 + trttime + scale(sex) + scale(Round) + scale(Order) + (1|Aviary) + (treat|ID), data=dat)

summary(mod5fig)

# (4) EPP ----------------------------------------------------------------------------------------

dat <- read.table(paste(path,"data_repsuccessNEW.txt",sep=""), sep="\t", header=TRUE)

mod6 <- glmer(epair ~ factor(Treat) + factor(Order) + factor(Round) + (1|gnest) + (1|gmother) + (1|aviary), data=dat, family="binomial")

summary(mod6)

sim.mod6 <- fixef(sim(mod6, n=2000))

quantile(sim.mod6[,1],c(0.025,0.975))

quantile(sim.mod6[,2],c(0.025,0.975))

quantile(sim.mod6[,3],c(0.025,0.975))

quantile(sim.mod6[,4],c(0.025,0.975))

mod6b <- lmer(scale(epair) ~ factor(Treat) + factor(Order) + factor(Round) + (1|gnest) + (1|gmother) + (1|aviary), data=dat)

summary(mod6b)

# (4) Reproductive success -----------------------------------------------------------------------

dat <- read.table(paste(path,"data_repsuccessNEW.txt",sep=""), sep="\t", header=TRUE)

# EM ---

mod7 <- glmer(F2 ~ factor(Treat) + factor(Order) + factor(Round) + (1|gmother) + (1|gfather) + (1|gpairID) + (1|aviary), data=dat, family="binomial")

summary(mod7)

sim.mod7 <- fixef(sim(mod7, n=2000))

quantile(sim.mod7[,1],c(0.025,0.975))

quantile(sim.mod7[,2],c(0.025,0.975))

quantile(sim.mod7[,3],c(0.025,0.975))

quantile(sim.mod7[,4],c(0.025,0.975))

mod7b <- lmer(scale(F2) ~ factor(Treat) + factor(Order) + factor(Round) + (1|gmother) + (1|gfather) + (1|gpairID) + (1|aviary), data=dat)

summary(mod7b)

# ChickMort

mod8 <- glmer(F3 ~ factor(Treat) + factor(Round) + (1|spairID) + (1|snest) + (1|aviary), data=dat, family="binomial")

summary(mod8) # makes no sense because only 7 dead chicks

sim.mod8 <- fixef(sim(mod8, n=2000))

quantile(sim.mod8[,1],c(0.025,0.975))

quantile(sim.mod8[,2],c(0.025,0.975))

quantile(sim.mod8[,3],c(0.025,0.975))

mod8b <- lmer(scale(F3) ~ factor(Treat) + factor(Round) + (1|spairID) + (1|snest) + (1|aviary), data=dat)

summary(mod8b)

# AdultOff

mod9 <- glmer(F6 ~ factor(Treat) + factor(Order) + factor(Round) + (1|gmother) + (1|gfather) + (1|gpairID)+ (1|aviary), data=dat, family="binomial")

summary(mod9) # almost identical to 1-EM

sim.mod9 <- fixef(sim(mod9, n=2000))

quantile(sim.mod9[,1],c(0.025,0.975))

quantile(sim.mod9[,2],c(0.025,0.975))

quantile(sim.mod9[,3],c(0.025,0.975))

quantile(sim.mod9[,4],c(0.025,0.975))

mod9b <- lmer(scale(F6) ~ factor(Treat) + factor(Round) + (1|gmother) + (1|gfather) + (1|gpairID)+ (1|aviary), data=dat)

summary(mod9b)

# ------------------------------------------------------------------------------------------------

**Supplemental literature cited**

Gelman, A. and Hill, J. (2007). Data analysis using regression and hierarchical/multilevel models: Cambridge University Press: Cambridge, UK.

Nakagawa, S. and Cuthill, I. C. (2007). Effect size, confidence interval and statistical significance: a practical guide for biologists. Biological Reviews 82, 591-605.
